# Dominant role of plant physiology in trend and variability of gross primary productivity in North America

**DOI:** 10.1038/srep41366

**Published:** 2017-02-01

**Authors:** Sha Zhou, Yao Zhang, Philippe Ciais, Xiangming Xiao, Yiqi Luo, Kelly K. Caylor, Yuefei Huang, Guangqian Wang

**Affiliations:** 1 State Key Laboratory of Hydroscience and Engineering, Department of Hydraulic Engineering, Tsinghua University, Beijing 100084, China; 2 Department of Civil and Environmental Engineering, Princeton University, Princeton, NJ 08544, USA; 3 Department of Microbiology and Plant Biology, Center for Spatial Analysis, University of Oklahoma, Norman, OK 73019, USA; 4 Laboratoire des Sciences du Climat et de l’Environnement, CEA CNRS UVSQ, Gif-sur-Yvette 91190, France; 5 Instittue of Biodiversity Science, Fudan University, Shanghai 200433, China; 6 Department of Microbiology and Plant Biology, University of Oklahoma, Norman, Oklahoma 73019, USA; 7 Center for Earth System Science, Tsinghua University, Beijing 100084, China; 8 College of Ecological and Environmental Engineering, Qinghai University, Xining 810086 Qinghai, China

## Abstract

Annual gross primary productivity (GPP) varies considerably due to climate-induced changes in plant phenology and physiology. However, the relative importance of plant phenology and physiology on annual GPP variation is not clear. In this study, a Statistical Model of Integrated Phenology and Physiology (SMIPP) was used to evaluate the relative contributions of maximum daily GPP (GPP_max_) and the start and end of growing season (GS_start_ and GS_end_) to annual GPP variability, using a regional GPP product in North America during 2000–2014 and GPP data from 24 AmeriFlux sites. Climatic sensitivity of the three indicators was assessed to investigate the climate impacts on plant phenology and physiology. The SMIPP can explain 98% of inter-annual variability of GPP over mid- and high latitudes in North America. The long-term trend and inter-annual variability of GPP are dominated by GPP_max_ both at the ecosystem and regional scales. During warmer spring and autumn, GS_start_ is advanced and GS_end_ delayed, respectively. GPP_max_ responds positively to summer temperature over high latitudes (40–80°N), but negatively in mid-latitudes (25–40°N). This study demonstrates that plant physiology, rather than phenology, plays a dominant role in annual GPP variability, indicating more attention should be paid to physiological change under futher climate change.

The importance of plant phenology shifts and physiology change on annual GPP variability is evident^1^,^2^,^3^,^4^. Warming-induced earlier leaf emergence enhances terrestrial carbon uptake in spring, whereas later leaf senescence in autumn also leads to a smaller increase in carbon uptake in North American temperate forests^5^. However, drought events associated with high temperature and low water availability can decrease plant photosynthetic uptake^6^,^7^,^8^. In regions exposed to summer drought, an increase of leaf area in earlier spring can accelerate soil drying, and lead to increased vulnerability of GPP in summer^9^,^10^. In terms of net carbon balance, carbon loss during summer drought can negate increased uptake in warmer springs and autumns^11^,^12^,^13^, related to the negative covariance between increased spring productivity and decreased yearly productivity. The different responses of plant phenology and physiology to climate anomalies, and the contributions of phenological and physiological changes to annual GPP variability must thus be disentangled.

The joint control of plant phenology and physiology on annual GPP can be expressed by constructing a statistical model^4^,^14^, which uses indicators to represent plant phenological and physiological changes. Phenology is about the time and duration of a process or event. The length of carbon uptake period (CUP), and the start (*GS*_*start*_) and the end (*GS*_*end*_) of the growing season, can be used as indicators of plant phenology. Plant photosynthesis is an important process of plant physiology, and can reflect the responses of plant physiology to environmental changes. As the maximum photosynthetic carbon uptake (*GPP*_*max*_) represents the important characteristics of plant photosynthesis, it can be used as an indicator of plant physiology. The variation in the product of CUP by *GPP*_*max*_ was found to explain more than 90% of the temporal variability of annual GPP in most areas of North America during 2000–2010, and more than 95% of the spatial GPP gradients among 213 flux tower sites^4^. This shows that even a simple statistical model has an interesting explanation power of observed variability of annual GPP. The Statistical Model of Integrated Phenology and Physiology (SMIPP) extends the approach of Xia *et al*.^4^ by treating separately *GS*_*start*_, *GS*_*end*_ and *GPP*_*max*_, as predictors of annual GPP. This model described in Zhou *et al*.^14^ was shown to explain 90 ± 11% of the inter-annual variability of GPP among 27 flux tower sites across North America and Europe. The SMIPP decomposes annual GPP anomaly into three components, which are induced by the anomalies in *GS*_*start*_, *GPP*_*max*_ and *GS*_*end*_, respectively. These three predictors were shown to be statistically independent of each other^14^, even though there may be processes linking them through lagged effects of previous season climate impacting the value of a predictor during the following seasons. Thus, the contributions of the changes in *GS*_*start*_, *GPP*_*max*_ and *GS*_*end*_ to annual GPP variability can be linked to the corresponding components of annual GPP anomaly.

Because the annual GPP variability is related to plant phenological and physiological changes, the responses of the above three indicators to climate variability is crucial to diagnose the drivers of annual GPP variability through the SMIPP. The sensitivity of plant phenology to climate change has been assessed in many studies^5^,^15^,^16^,^17^,^18^. While spring phenology is unambiguously advanced during warmer springs, autumn phenology is affected not only by temperature, but also by precipitation, photoperiod, and cooling degree-days^19^,^20^,^21^,^22^,^23^. In addition, the climatic responses of plant phenology vary among different species and climate regions^24^,^25^,^26^. Although the impacts of climate change on *GPP*_*max*_ are more difficult to assess, summer photosynthesis is usually reduced by hot temperatures associated with dryer soils^9^,^13^,^27^. Because plant “phenology” (here *GS*_*start*_ and *GS*_*end*_) and “physiology” (here *GPP*_*max*_) are influenced by climatic factors in different ways, it is important to investigate how plant phenology and physiology mediate impacts of climate variability on annual GPP variability through their responses to climate variability.

In this study, we evaluate the relative contributions of the changes in *GS*_*start*_, *GPP*_*max*_ and *GS*_*end*_ to the long-term trend and inter-annual variability of GPP using the SMIPP. Our study is based on a regional GPP product from the vegetation photosynthesis model (VPM) in North America over the period 2000–2014. The relative contributions of the three indicators are also investigated at ecosystem scale based on GPP measurements from 24 AmeriFlux sites. In addition, we derive the climatic sensitivity of the three indicators, and investigate the mechanism of annual GPP responses to temperature, precipitation and downward solar radiation through plant phenological and physiological changes over different regions.

## Results

### Long-term trend of annual GPP and the three indicators

**Figure 1:**
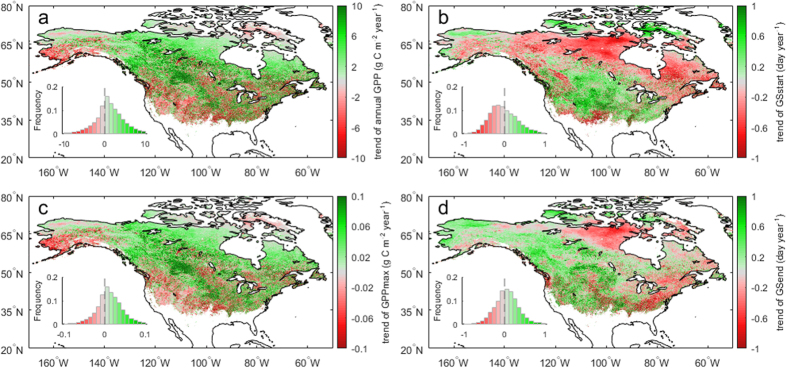
15-year (2000–2014) trends of annual GPP and the three indicators in North America. (**a**) Annual GPP. (**b**) Start of growing season (*GS*_*start*_). (**c**) Maximum daily GPP (*GPP*_*max*_). (**d**) End of growing season (*GS*_*end*_). A negative sign (red color) in (**b**) and (**d**) denotes an earlier trend of *GS*_*start*_ and *GS*_*end*_, and vice versa. Maps were generated using MATLAB 2015b (http://www.mathworks.com/products/matlab/).

Figure 1a shows the spatial pattern of the long-term trend of annual GPP from VPM over mid- and high latitudes in North America during the period 2000–2014. Annual GPP trend ranges from −10 to 10 g C m^−2^ per year for 96.7% of the area, and the average rate is 1.40 g C m^−2^ per year over the whole area. The majority (66.5% of the area) experienced a positive trend in annual GPP with an increasing rate of 3.47 ± 3.36 g C m^−2^ per year. However, other regions experienced a negative trend (−2.72 ± 2.91 g C m^−2^ per year) in annual GPP. Regions with decreasing GPP are mainly located in the western US and south Alaska.

Earlier *GS*_*start*_ is evident across the boreal zone, while most of the temperate zone show delayed *GS*_*start*_ (Fig. 1b, see Fig. S1 for the climate zones over North America). The average advance of *GS*_*start*_ is 0.29 day per year for 57% of the area, and the average delay is 0.32 day per year for the other area. *GS*_*end*_ delays by 0.26 day per year for 58% of the area, most of which are located at the western and central North America (Fig. 1d). Spatial correlations were performed using the values of the long-term trends of annual GPP and the three indicators from all the grid cells. The spatial correlation between the long-term trends of either *GS*_*start*_ or *GS*_*end*_ and annual GPP is weak (R^2^ < 0.10). However, both the mean value (R^2^ = 0.83, p < 0.001), and the trend of *GPP*_*max*_ (R^2^ = 0.90, p < 0.001) exhibit a nearly identical spatial pattern with annual GPP (Fig. S2a,c and Fig. 1a,c). The high spatial correlations of the mean values and trends between *GPP*_*max*_ and annual GPP indicate the importance of *GPP*_*max*_ in annual GPP and the major contribution of *GPP*_*max*_ trend to annual GPP trend.

### Annual GPP variability explained by the SMIPP

**Figure 2:**
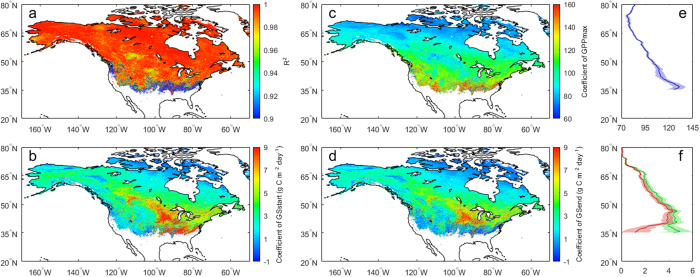
Multiple regression results of the statistical model of integrated phenology and physiology (SMIPP). (**a**) R^2^ of the SMIPP. Sensitivity coefficients of annual GPP to (**b**) *GS*_*start*_, (**c**) *GPP*_*max*_, and (**d**) *GS*_*end*_. Latitudinal distributions of the sensitivity coefficients of annual GPP to (**e**) *GPP*_*max*_ (purple), and (**f**) *GS*_*start*_ (green) and *GS*_*end*_ (red). Maps were generated using MATLAB 2015b (http://www.mathworks.com/products/matlab/).

First of all, the interrelationships of the three indicators were tested. R^2^ is 0.26 ± 0.23 between *GS*_*start*_ and *GPP*_*max*_ (p > 0.05 over 56.8% of the area), 0.11 ± 0.13 between *GPP*_*max*_ and *GS*_*end*_ (p > 0.05 over 86.9% of the area), and 0.23 ± 0.20 between *GS*_*start*_ and *GS*_*end*_ (p > 0.05 over 62.7% of the area), respectively, across all the grid cells (Fig. S4). Our correlation analysis indicates the independence of the three indicators over most study area. The SMIPP was then applied to each grid cell and the regression results are provided in Fig. 2. The SMIPP can explain 98.3 ± 4.7% of inter-annual variability of the GPP from VPM across mid- and high latitudes in North America (p < 0.001 over 99.1% of the area (Fig. S5a)). The model is robust over temperate, boreal and tundra climate zones, and a little weak (R^2^ < 0.90) in subtropical and Mediterranean climate zones over southeast and west coast of the US.

The data displayed in Fig. 2c and e show that annual GPP changes are less sensitive to change in *GPP*_*max*_ over higher latitudes. An increase of 1 g C m^−2^ day^−1^ in *GPP*_*max*_ contributes an increase of 95.9 ± 17.3 g C m^−2^ per year in annual GPP across the study area, but the value of the sensitivity of annual GPP to *GPP*_*max*_ decreases from South to North, from 125 around 35°N to 80 around 70°N. The sensitivities of annual GPP to *GS*_*start*_ and *GS*_*end*_ follow a similar pattern over North of 45°N. However, their sensitivity coefficients gradually diverge from 45 to 35°N because annual GPP is more sensitive to *GS*_*start*_ than to *GS*_*end*_ over eastern subtropical coasts of the US. Overall, the sensitivity of annual GPP relative to *GS*_*start*_ (2.9 ± 2.2 g C m^−2^ day^−1^) is a little higher than that relative to *GS*_*end*_ (2.4 ± 1.8 g C m^−2^ day^−1^). The sensitivity coefficient of annual GPP to *GPP*_*max*_ is significant at 0.001 level over 99.3% of the area and that to *GS*_*start*_ and *GS*_*end*_ is significant at 0.01 level over 89.2% and 85.2% of the area, respectively (Fig. S5). Thus, the three sensitivity coefficients can capture the sensitivity of annual GPP to *GS*_*start*_, *GPP*_*max*_, and *GS*_*end*_, respectively, over mid- and high latitudes in North America.

### Contributions of the three indicators to annual GPP variability

**Figure 3:**
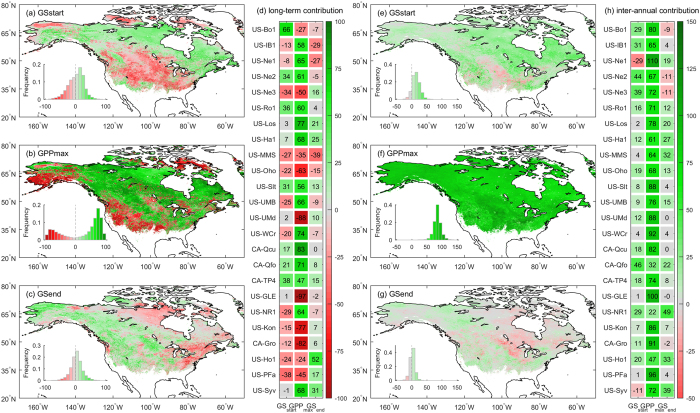
Relative contributions (%) of the three indicators to annual GPP variability in North America. Contributions of (**a**,**e**) *GS*_*start*_, (**b**,**f**) *GPP*_*max*_, and (**c**,**g**) *GS*_*end*_ to the long-term trend and inter-annual variability in annual GPP, respectively. Contributions of the three indicators to the (**d**) long-term trend and (**h**) inter-annual variability in annual GPP for the 24 AmeriFlux sites. Maps were generated using MATLAB 2015b (http://www.mathworks.com/products/matlab/).

The contributions of the three indicators to the long-term trend in annual GPP were separated based on the results of the SMIPP (Fig. 3a–c). The contribution is positive only when the indicator and annual GPP have a trend of the same sign, and vice versa. The *GPP*_*max*_ related component of GPP, contributes positively to annual GPP increase over 67.4% of the area, and negatively over other areas, such as the western US and south Alaska area, where annual GPP shows decreasing trends. The positive and negative contributions are 65.4 ± 20.3% and 61.2 ± 24.7%, respectively. In addition, *GPP*_*max*_ contributes more than half of annual GPP change for 77.1% of the area (54.3% positive and 22.8% negative), indicating that the long-term trend in annual GPP is dominated by *GPP*_*max*_ trend. By comparison, the positive and negative contributions are 20.0 ± 14.0% and 21.9 ± 17.3% for *GS*_*start*_, and only 15.8 ± 14.0% and 14.4 ± 13.7% for *GS*_*end*_. The primary contribution of *GPP*_*max*_ and the secondary contributions of *GS*_*start*_ and *GS*_*end*_ are in agreement with the spatial correlation results between the long-term trends of annual GPP and the three indicators.

The inter-annual variability of GPP is also dominated by the variability of the *GPP*_*max*_ related component, whose contribution accounts for 84.6 ± 14.7%, and for more than 50% over 98.8% of the area (Fig. 3e–g). The contribution of *GS*_*start*_ is 13.8 ± 13.70%, with positive values in 87.9% of the area. In most of the northern plains area, *GS*_*start*_ varies oppositely with annual GPP and contributes negatively to annual GPP variability. *GS*_*end*_ explains the least of the inter-annual variability of GPP, and its contribution is as low as 1.6 ± 8.7%, ranging only from −15% to 15% for more than 90% of the area. Thus, *GS*_*start*_ plays a more important role than *GS*_*end*_ in terms of its contribution to the long-term trend and inter-annual variability of GPP.

The dominant contribution of the *GPP*_*max*_ related component of GPP to the long-term trend and inter-annual variability of GPP is also supported by the AmeriFlux data. The SMIPP was calibrated at 24 flux sites with the R^2^ of 0.94 ± 0.05 (Table S2). The positive contribution of *GPP*_*max*_ to the long-term trend of annual GPP is observed at 14 of the 24 sites with mean contribution of 65.4%, and the mean negative contribution is 58.7% (Fig. 3d). In addition, *GPP*_*max*_ contributes 74.2 ± 19.7% to the inter-annual variability of GPP over the 24 sites (Fig. 3h). The attribution analyses consistently demonstrate the dominant role of *GPP*_*max*_ in the long-term trend and inter-annual variability of GPP across mid- and high latitudes in North America.

### Climatic sensitivity of the three indicators and of annual GPP

**Figure 4:**
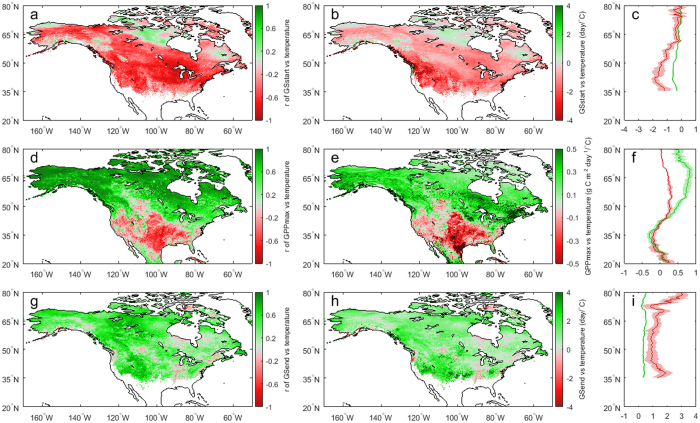
Correlation coefficient (r) and slope between the three indicators and respective seasonal temperature in North America. (**a**,**b**) *GS*_*start*_ and preseason (30 days before the mean date of *GS*_*start*_) temperature. (c, d) *GPP*_*max*_ and summer temperature (mean temperature in June-July-August). (**e**,**f**) *GS*_*end*_ and preseason (30 days before the mean date of *GS*_*end*_) temperature. Maps were generated using MATLAB 2016a (http://www.mathworks.com/products/matlab/).

The responses of the three indicators to the changes in temperature, precipitation and solar radiation were investigated. Both *GS*_*start*_ and *GS*_*end*_ are strongly related to preseason (30 days before the mean dates of *GS*_*start*_ and *GS*_*end*_, respectively) temperature (r = −0.48 ± 0.24 for *GS*_*start*_ and r = 0.32 ± 0.26 for *GS*_*end*_) in most study area (Fig. 4a and e). *GS*_*start*_ occurs 1.9 ± 1.4 days earlier, and *GS*_*end*_ occurs 1.7 ± 1.8 days later, with an increase of 1 °C in preseason temperature (Fig. 4b and f). Earlier *GS*_*start*_ and later *GS*_*end*_ in response to warmer temperature lead to more carbon assimilation in spring and autumn, especially over mid-latitudes, where annual GPP shows higher sensitivity to *GS*_*start*_ and *GS*_*end*_ than in high latitudes (Fig. 2). It appears that higher preseason precipitation delays *GS*_*start*_ and advances *GS*_*end*_, respectively, over most areas (Fig. S6). However, the correlation of *GS*_*start*_ or *GS*_*end*_ with precipitation is weaker than that with temperature (Figs 4 and S6). In addition to temperature, *GS*_*end*_ is also delayed by higher preseason solar radiation over 81.7% of the study area, although its correlation with solar radiation is a little weaker (r = 0.27 ± 0.28) (Fig. S7). *GS*_*start*_ is weakly correlated with preseason solar radiation over most study area, suggesting that *GS*_*start*_ is mainly affected by temperature, which is also supported by the partial correlation analysis (Fig. S8).

**Figure 5:**
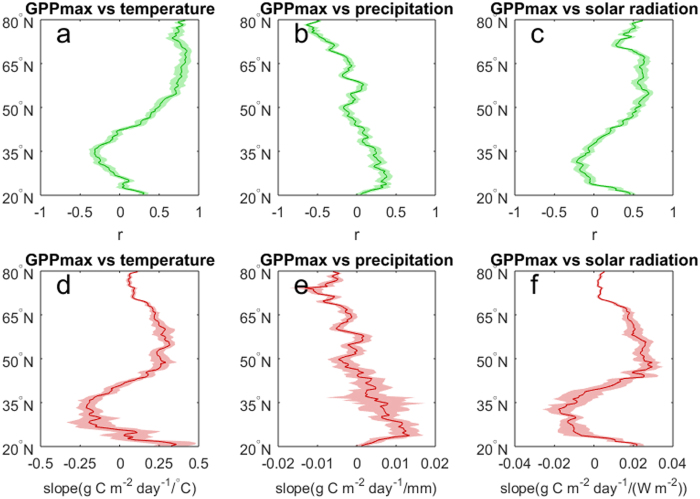
Latitudinal distributions of r and slope for the relationships between *GPP*_*max*_ and climatic factors. (**a**,**d**) *GPP*_*max*_ v.s. summer temperature (mean temperature in June-July-August). (**b**,**e**) *GPP*_*max*_ v.s. summer precipitation (mean monthly precipitation in June-July-August). (**c**,**f**) *GPP*_*max*_ v.s. summer solar radiation (mean solar radiation in June-July-August).

*GPP*_*max*_ presents opposite responses to summer temperature over different regions. *GPP*_*max*_ is greatly enhanced in high latitudes (40–80°N) while reduced in mid-latitudes (25–40°N) with warmer summer temperature (Fig. 4c and d). The sensitivity in *GPP*_*max*_ to higher summer temperature increases from less than 0.1 g C m^−2^ day^−1^/°C around 80°N to 0.33 g C m^−2^ day^−1^/°C around 60°N and decreases to −0.2 g C m^−2^ day^−1^/°C around 35°N (Fig. 5d). Although earlier *GS*_*start*_ and later *GS*_*end*_ enhance carbon uptake, the declining *GPP*_*max*_ with higher temperature would probably decrease summer GPP and even cancel out the enhanced spring and autumn GPP, resulting in annual GPP decline over most of the great plain areas (Fig. S9a and b). On the contrary, warmer temperature has a positive impact on annual GPP anomaly in the boreal forests where advanced *GS*_*start*_, increased *GPP*_*max*_ and delayed *GS*_*end*_ during warmer spring, summer, autumn consistently contribute to increasing annual GPP (Fig. S9a and b). Similarly, *GPP*_*max*_ responds positively to higher summer solar radiation in high latitudes, while negatively in most mid-latitudes, especially the great plain areas (Fig. S7c and d). It is worth noting that the spatial pattern of the sensitivity in *GPP*_*max*_ to summer precipitation is different from that to summer temperature and solar radiation, positively in mid-latitudes (central US) and negatively in most high-latitudes (Fig. S6c and d). The significant trends of rising temperature and higher solar radiation in summer strongly enhance carbon assimilation in high latitudes for the period 2000–2014 (Figs 1a and S10a and c), and imply more carbon to be assimilated under future climate warming. The recent increasing trend of summer precipitation, will partially relieve the stress of high temperature and solar radiation, and accelerate summer GPP in mid-latitudes (Fig. S10a–c).

## Discussion

In this study, we found that plant physiology plays a more important role than phenology in determining the long-term trend and inter-annual variability of VPM GPP and site scale AmeriFlux GPP. The importance of plant phenology on seasonal and annual GPP variability has been shown in many studies^1^,^2^,^5^,^28^,^29^. However, phenological changes cannot explain GPP reduction caused by the climate extreme events, which account for the majority of global inter-annual variability in GPP^30^,^31^. Because of the direct link between photosynthetic physiology and carbon assimilation, *GPP*_*max*_ used in this study is strongly correlated to annual GPP and contributes to most of the inter-annual variability of GPP. The results of Xia *et al*.^4^ also indicated that the contribution of *GPP*_*max*_ is larger than CUP to the spatial variability of GPP over most biome types, based on partial correlation analysis^4^. Given the dominant role of *GPP*_*max*_ in the long-term trend and inter-annual variability of GPP, more focus should be paid to plant physiological change to better explain GPP variability and improve GPP monitor in terrestrial ecosystems^32^.

The plant phenological and physiological changes were related to temperature, precipitation, and solar radiation. The temperature control of *GS*_*start*_ is stronger than the effects of precipitation and solar radiation over most study area. Since bud burst depends on accumulated temperature, and plant photosynthesis is also temperature dependent, preseason temperature is responsible for triggering phenological events. Many studies also indicated that the phenology of the boreal and temperate forests is manly driven by temperature^5^,^15^,^33^. Apart from temperature, *GS*_*end*_ is also regulated by preseason solar radiation. The solar radiation is typically not limiting when air temperature triggers the onset of plant photosynthesis in spring^34^. However, higher solar radiation can delay leaf senescence in autumn. For one thing, the accumulation of abscisic acid which is responsible for leaf senescence can be inhibited by higher solar radiation; for the other, the plant photosynthetic capacity is enhanced by higher solar radiation, resulting in higher photosynthetic rate and later leaf senescence^19^.

In comparison with the consistent temperature responses of *GS*_*start*_ and *GS*_*end*_ over most of the area, *GPP*_*max*_ responds positively to summer temperature in high latitudes, while negatively in mid-latitudes, where *GPP*_*max*_ is mainly enhanced by summer precipitation. The opposite responses of *GPP*_*max*_ to summer temperature between mid- and high latitudes can support the simulation results that global warming would increase the productivity at northern high-latitudes but tend to reduce it in the mid-latitudes and tropics^35^. The inter-annual variability of tree ring width in Europe is also mainly controlled by temperature in high latitudes while by precipitation (or water availability) in mid-latitudes based on the radial tree growth analysis^36^, but tree ring data cannot be easily related to climate variable during a specific season, given species-specific onthogenic controls of cambial wood growth during periods of the growing season. The latitudinal patterns of the temperature sensitivity of *GS*_*start*_ and *GS*_*end*_ and the opposite temperate sensitivity of *GPP*_*max*_ over mid- and high latitudes are very important in predicting the phenological and physiological responses to climate change.

Recent climate warming seems to have a generally positive impact on forest productivity when water is not limiting^37^. Because the boreal forests are mainly limited by sunlight and temperature, and the temperate forests are mainly limited by water^38^, *GPP*_*max*_ tends to increase in high latitudes and decrease in mid-latitudes with increasing temperature. The negative response of *GPP*_*max*_ to the summer temperature over most mid-latitudes of North America indicates a decreasing strength of temperate and sub-tropical ecosystems in carbon assimilation under recent warming. Although the advanced *GS*_*start*_ and delayed *GS*_*end*_ can compensate part of *GPP*_*max*_ induced GPP decline, annual GPP is still negatively correlated with annual temperature over the Central Great Plains in the US (Fig. S9a and b), where annual productivity is highly limited by water. The warming trend seems to slow down in recent years, however, the projected North America temperature will continue increasing over the next several decades^39^,^40^. Thus, warming induced GPP decline is expected in part of the low and mid-latitudes in the future.

The results indicate that advanced *GS*_*start*_, increased *GPP*_*max*_ and delayed *GS*_*end*_ under warming climate consistently contribute to increase annual mean GPP in high latitudes, but increasing GPP will not necessarily result in an increase in net ecosystem productivity (NEP). It was reported that the growth of western North American boreal forests is constrained by both the increasing costs of autotrophic respiration^41^,^42^ and frequent summer drought^9^,^12^,^43^,^44^, because higher temperature and longer growing season will accelerate greater ecosystem respiration and may exacerbate water stress in summer. However, much larger seasonal CO_2_ amplitude change was found over north of 45°N, which is mainly due to summer carbon uptake, than that for 10 to 45°N from 1960 s to 2010 s in the North Hemisphere^45^, indicating that summer NEP was more greatly enhanced at boreal than temperate zones over the 50-year period. Although the high potential of carbon assimilation in North American high latitude ecosystems may be offset by the warming induced high respiration and drought stress in some regions, the boreal forest ecosystems will continue to play an important role in sequestering atmospheric CO_2_ during the growing season under warming climate.

This study shows that the long-term trend and inter-annual variability of GPP is dominated by *GPP*_*max*_ based on both VPM GPP and data from 24 AmeriFlux sites. Although *GS*_*start*_ and *GS*_*end*_ also exert strong control over annaul GPP, their contributions to annual GPP change are much weaker than *GPP*_*max*_, indicating great importance of physiological change on annual GPP variability under changing climate. The consistently positive responses of the three indicators to warming temperature reveal that annual carbon assimilation will benefit from the warming climate in high latitudes, although the ecosystem respiration and drought stress may reduce the net ecosystem productivity in some regions; on the contrary, the divergent responses of plant phenology and physiology to the warming temperature reduce annual carbon uptake over part of low and mid-latitudes in North America.

## Methods

### Data sets

A regional GPP product in North America from 2000 to 2014 was produced using the vegetation photosynthesis model (VPM)^46^,^47^. VPM is a production efficiency model which uses the product of the photosynthetically active radiation absorbed by chlorophyll and a light use efficiency factor to estimate GPP^48^. The regional VPM GPP product has been validated using both flux tower data and the solar-induced chlorophyll fluorescence (SIF) data from the Global Ozone Monitoring Experiment-2 (GOME-2) across North America^46^. The VPM GPP agrees well with flux tower derived GPP at 39 AmeriFlux sites (R^2^ = 0.82 for all sites) and shows good consistency with the GOME-2 SIF data in terms of spatial distribution and seasonal dynamics^46^. The three indicators were derived from the 8-day 0.05 degree VPM GPP product for each grid cell in this study over the period 2000–2014 across North America.

The three indicators were also derived at ecosystem scale using the eddy covariance data from 24 AmeriFlux sites (Table S1). There are six vegetation types among the 24 sites, including Deciduous Broadleaf Forest (DBF), Evergreen Needle-leaf Forest (ENF), Mixed Forest (MF), Cropland (CRO), Grassland (GRA), Closed Shrub Land (CSH). Most of the sites are located at temperate climate zones, and only two ENF sites located at boreal climate zones (Fig. S1). The data records at individual sites range from 6 to 21 years, and there are 253 site-years in total. Half-hourly GPP estimates were derived from net ecosystem exchange measurements, which were gap-filled and partitioned using the R package ‘REddyProc’ (http://r-forge.r-project.org/R/?group_id=1679), following the method of Reichstein *et al*.^49^. The half-hourly GPP was aggregated to daily GPP time series to derive the three indicators for each site year.

The temperature, precipitation and downward solar radiation data used for sensitivity analysis are from the National Center for Environmental Prediction-North American Regional Reanalysis (NARR) products^50^. Daily NARR temperature, precipitation and solar radiation data at 32 km spatial resolution were spatially interpolated into 0.05 degree and then temporally aggregated to obtain the seasonal and annual data. The climatic data during the preseason period, i.e., 30 days preceding the mean dates of *GS*_*start*_ and *GS*_*end*_ during 2000–2014, were calculated for climatic sensitivity analyses. Since *GPP*_*max*_ occurred in June-August for more than 99.9% of the study area (Fig. S3), we used the climatic data in summer (June-July-August) to evaluate the climatic sensitivity of *GPP*_*max*_. Annual GPP was correlated to annual temperature, precipitation and solar radiation to investigate the climatic sensitivity of annual GPP. In view of the strong relationships between *GS*_*start*_ and spring GPP, *GPP*_*max*_ and summer GPP, and *GS*_*end*_ and autumn GPP^14^, the climatic sensitivity of annual GPP can be better understood by combining the responses of the three indicators to their respective seasonal climatic factors.

### Indicator identification

The three indicators, *GS*_*start*_, *GPP*_*max*_ and *GS*_*end*_, were determined from the smoothed time series of GPP for each year. In case of some abnormal values, the singular spectrum analysis was performed to derive smoothed daily GPP curves for the 253 site-years. The “Rssa” package (https://cran.r-project.org/web/packages/Rssa/index.html) in R was used to obtain smoothed daily GPP curve. First, the daily GPP series were decomposed into new time series which consist of different frequency components and noises according to the singular value decomposition. Second, the seasonal signal, i.e., the smoothed daily GPP, was reconstructed from the decomposed components. In this study, we used the first four components corresponding to the lowest frequency sub-signals to derive the smoothed daily GPP curve from the original time series.

In terms of the VPM GPP product, a least-square regression analysis was performed between the 8-day GPP and the corresponding day of year (DOY) for the whole year. We used a sixth-degree polynomial function to fit the seasonal GPP curve as a function of DOY^15^


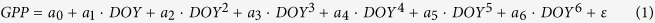


where *a*_*i*_(*i* = 0~6) are the fitted parameters and *ε* is the error term. Based on the equation (1), a smoothed GPP curve can be constructed for the determination of the three indicators. *GPP*_*max*_ was determined as the peak value of the smoothed GPP curve. *GS*_*start*_ and *GS*_*end*_ were identified as the first and last days when the smoothed GPP crossed a given threshold. In this study, the threshold was set to be 10% of the long-term average *GPP*_*max*_ over all the available years for each site and over 2000–2014 for each grid cell^14^.

### The SMIPP model

The three indicators, *GS*_*start*_, *GPP*_*max*_, and *GS*_*end*_, are involved in the SMIPP to represent the phenological and physiological impacts on annual GPP, and their contributions to annual GPP change are seperated based on a total differential function. Firstly, annual GPP is expressed as a function of the three indicators, that is


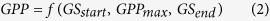


Assuming the three indicators are independent of one another, the total differential of GPP with respect to all the three indicators is


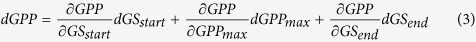


Since the three partial derivatives denote the sensitivity of annual GPP with respect to the changes in the three indicators, we use three sensitivity coefficients *η*_*start*_, 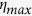, 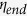 to represent the three partial derivatives. In practice, the differentials of annual GPP and of the three indicators are approximated by the anomalies (Δ) of the variables, namely, the differences between the variables with respect to their long-term mean values. Thus, the equation (3) transforms into


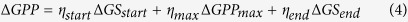


To make the anomalies of *GS*_*start*_ easier to compare with those of *GS*_*end*_, anomalies are by convention, counted positive when *GS*_*start*_ advances and when *GS*_*end*_ delays relative to their long-term mean values. With the observed anomalies of annual GPP and the three indicators, the three sensitivity coefficients can be estimated based on a multiple regression for each grid cell of the VPM GPP product or of GPP observed at each flux tower site. Thus, the annual GPP anomaly is separated into three independent components, i.e., *η*_*start*_Δ*GS*_*start*_, *η*_*max*_Δ*Gpp*_*max*_, *η*_*end*_Δ*GS*_*end*_, representing the annual GPP change induced by the three indicators, respectively.

### Attribution analysis

The relative contributions of the changes in the three indicators to the long-term trend and inter-annual variability were calculated. As shown in equation (4), annual GPP anomaly consists of three independent components, thus, the long-term trend of annual GPP can also be separated into three independent trends, expressed by *Slope*_*start*_, *Slope*_*max*_ and *Slope*_*end*_. Because the three indicators may contribute positively or negatively to the long-term trend of annual GPP, the relative contributions of them were calculated as the ratios of *Slope*_*start*_, *Slope*_*gpp*_, *Slope*_*end*_ over the total absolutes of these three slopes.


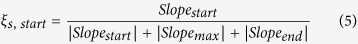



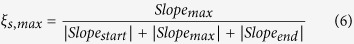



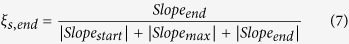


where 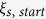, 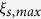, and 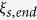 represent the relative contributions of the trends of the three indicators related components of GPP to the long-term linear trend of annual GPP. In equations (5),(6),(7), a positive sign denotes an increasing trend of annual GPP contributed by the corresponding indicator, and vice versa, and the magnitude denotes the amount of the relative contribution.

The relative contributions of the changes in the three indicators to the inter-annual variability of GPP were calculated according to the consistency of 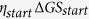, 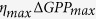, 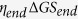 with annual GPP anomaly over the period 2000–2014^51^.


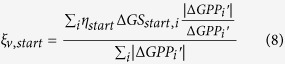



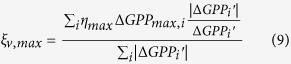



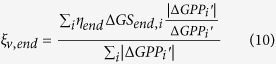


where *i* refers to the year from 2000 to 2014, and 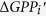 is the estimated annual GPP anomaly based on the SMIPP. The 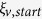, 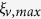, and 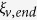 represent the relative contributions of the three indicators related components of GPP to the inter-annual variability of GPP. In equations (8),(9),(10), the positive sign reveals identical inter-annual variability of the indicator with annual GPP, and vice versa, and the magnitude denotes the amount of the relative contribution.

## Additional Information

**How to cite this article**: Zhou, S. *et al*. Dominant role of plant physiology in trend and variability of gross primary productivity in North America. *Sci. Rep.* **7**, 41366; doi: 10.1038/srep41366 (2017).

**Publisher's note:** Springer Nature remains neutral with regard to jurisdictional claims in published maps and institutional affiliations.

## Supplementary Material

Supplementary Information
