# Supplementary material for: Dominant role of plant physiology in trend and variability of gross primary productivity in North America
Source: Sci Rep. 2017 Feb 1;7:41366. doi: 10.1038/srep41366 (PMC5286448; doi:10.1038/srep41366)
Supplement: Supplementary Information [file srep41366-s1.pdf]

Supplementary Information for

Dominant role of plant physiology in trend and variability of gross primary productivity in North America

Sha Zhou[1,2], Yao Zhang[3], Philippe Ciais[4], Xiangming Xiao[3,5], Yiqi Luo[6,7], Kelly K. Caylor[2], Yuefei Huang[1,8], Guangqian Wang[1]

[1]State Key Laboratory of Hydroscience and Engineering, Department of Hydraulic Engineering, Tsinghua University, Beijing 100084, China

[2]Department of Civil and Environmental Engineering, Princeton University, Princeton, NJ 08544, USA

[3]Department of Microbiology and Plant Biology, Center for Spatial Analysis, University of Oklahoma, Norman, OK 73019, USA

[4]Laboratoire des Sciences du Climat et de l'Environnement, CEA CNRS UVSQ, Gif-sur-Yvette 91190, France

[5]Instittue of Biodiversity Science, Fudan University, Shanghai 200433, China

[6]Department of Microbiology and Plant Biology, University of Oklahoma, Norman, Oklahoma 73019, USA

[7]Center for Earth System Science, Tsinghua University, Beijing 100084, China

[8]College of Ecological and Environmental Engineering, Qinghai University, Xining 810086 Qinghai, China

Correspondence to: Sha Zhou (zhous13@mails.tsinghua.edu.cn) and Yuefei Huang (yuefeihuang@tsinghua.edu.cn)

Table S1. Twenty four eddy covariance sites in North America used in this study. Site descriptions include Site Identifier (ID), Plant Functional Type (PFT), Latitude (Lat, °N), Longitude (Lon, °W), Period of Record, and a reference for each sites. PFTs were taken from the International Geosphere-Biosphere Program (IGBP) land cover classification scheme (DBF = Deciduous Broadleaf Forest, ENF = Evergreen Needle Leaf Forest, MF = Mixed Forest, CRO = Cropland, GRA = Grassland, CSH = Closed Shrub Land).

site	PFT	Lat	Long	Period of record	reference
US-Bo1	CRO	40.0062	-88.2904	1997-1999,2000-2007	1
US-IB1	CRO	41.8593	-88.2227	2005-2011	2
US-Ne1	CRO	41.1650	-96.4766	2001-2012	3
US-Ne2	CRO	41.1649	-96.4701	2001-2012	3
US-Ne3	CRO	41.1797	-96.4396	2001-2012	3
US-Ro1	CRO	44.7143	-93.0898	2004-2009,2011-2012	4
US-Los	CSH	46.0827	-89.9792	2001-2008,2010,2014	5
US-Ha1	DBF	42.5378	-72.1715	1992-2012	6
US-MMS	DBF	39.3231	-86.4131	1999-2014	7
US-Oho	DBF	41.5545	-83.8438	2004,2006-2013	8
US-Slt	DBF	39.9137	-74.5960	2005-2012	9
US-UMB	DBF	45.5598	-84.7138	2005-2014	10
US-UMd	DBF	45.5625	-84.6975	2007-2014	11
US-WCr	DBF	45.8060	-90.0798	1999-2006,2011-2014	12
CA-Qcu	ENF	49.2671	-74.0365	2002-2009	13
CA-Qfo	ENF	49.6925	-74.3420	2003-2009	14
CA-TP4	ENF	42.7098	-80.3574	2003-2012	15
US-GLE	ENF	41.3644	-106.2394	2005-2012	16
US-NR1	ENF	40.0329	-105.5464	2000-2013	17
US-Kon	GRA	39.0824	-96.5603	2007-2012	18
CA-Gro	MF	48.2174	-82.1555	2004-2013	19
US-Ho1	MF	45.2041	-68.7402	1996-2004,2006-2008	20
US-PFa	MF	45.9459	-90.2723	1997-2004,2006-2014	21
US-Syv	MF	46.2420	-89.3477	2002-2006,2014	22

Table S2. The estimated physiological and phenological sensitivity coefficients, i.e., η_{start} (g C m^{-2} day^{-1}), η_{gpp} (g C m^{-2} per year/g C m^{-2} day^{-1}), and η_{end} (g C m^{-2} day^{-1}), and the coefficient of determination (R^2) of the SMIPP for each of the 24 sites. The statistically significant (p-value) of the regression equations and sensitivity coefficients are shown as well.

site	PFT	R^2	p-value	η_{start}	p-value	η_{gpp}	p-value	η_{end}	p-value
US-Bo1	CRO	0.94	<0.001	6.60	0.175	71.12	0.001	6.20	0.558
US-IB1	CRO	0.99	<0.001	8.13	0.012	72.59	0.002	7.75	0.020
US-Ne1	CRO	0.99	<0.001	13.19	<0.001	82.81	<0.001	12.81	<0.001
US-Ne2	CRO	1.00	<0.001	14.27	<0.001	57.22	<0.001	13.92	<0.001
US-Ne3	CRO	1.00	<0.001	12.07	<0.001	59.15	<0.001	10.92	<0.001
US-Ro1	CRO	1.00	<0.001	7.75	0.001	57.00	<0.001	13.09	<0.001
US-Los	CSH	0.97	<0.001	4.85	0.010	72.24	<0.001	5.16	0.004
US-Ha1	DBF	0.93	<0.001	8.23	<0.001	78.66	<0.001	11.13	0.027
US-MMS	DBF	0.87	<0.001	5.67	0.011	134.37	<0.001	7.84	<0.001
US-Oho	DBF	0.94	0.001	11.64	0.006	83.76	0.001	10.70	0.006
US-Slt	DBF	0.91	0.005	4.57	0.534	107.79	0.003	7.49	0.606
US-UMB	DBF	0.99	<0.001	8.01	<0.001	71.92	<0.001	6.59	<0.001
US-UMd	DBF	1.00	<0.001	8.67	<0.001	89.43	<0.001	3.54	<0.001
US-WCr	DBF	0.97	<0.001	7.17	<0.001	77.42	<0.001	4.38	0.175
CA-Qcu	ENF	1.00	<0.001	1.76	0.007	94.25	<0.001	0.09	0.872
CA-Qfo	ENF	0.91	0.015	3.67	0.045	101.47	0.026	3.06	0.052
CA-TP4	ENF	0.82	0.005	3.30	0.168	93.08	0.003	3.08	0.527
US-GLE	ENF	0.94	0.002	0.57	0.732	151.92	<0.001	-0.12	0.935
US-NR1	ENF	0.87	<0.001	3.23	0.013	92.95	0.001	6.07	0.000
US-Kon	GRA	0.98	0.005	4.27	0.116	120.23	0.002	5.32	0.136
CA-Gro	MF	0.92	<0.001	5.52	0.008	107.83	0.000	-1.14	0.489
US-Ho1	MF	0.89	<0.001	4.74	0.024	112.43	0.001	4.28	0.011
US-PFa	MF	0.84	<0.001	4.06	0.066	72.18	<0.001	1.60	0.215
US-Syv	MF	0.94	0.025	10.09	0.153	72.63	0.029	9.47	0.105

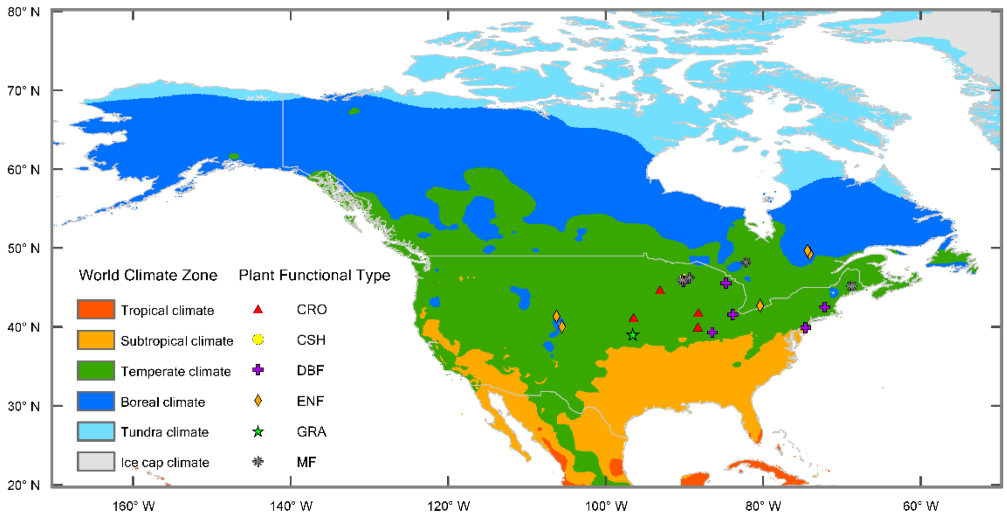

Figure S1. Climate zones over North America and the locations of the 24 AmeriFlux sites. The world climate zones used is based on the updated world map of the Koppen-Geiger climate classification[23]. The map was generated using ArcGIS 10.3 (http://www.esri.com/software/arcgis/arcgis-for-desktop).

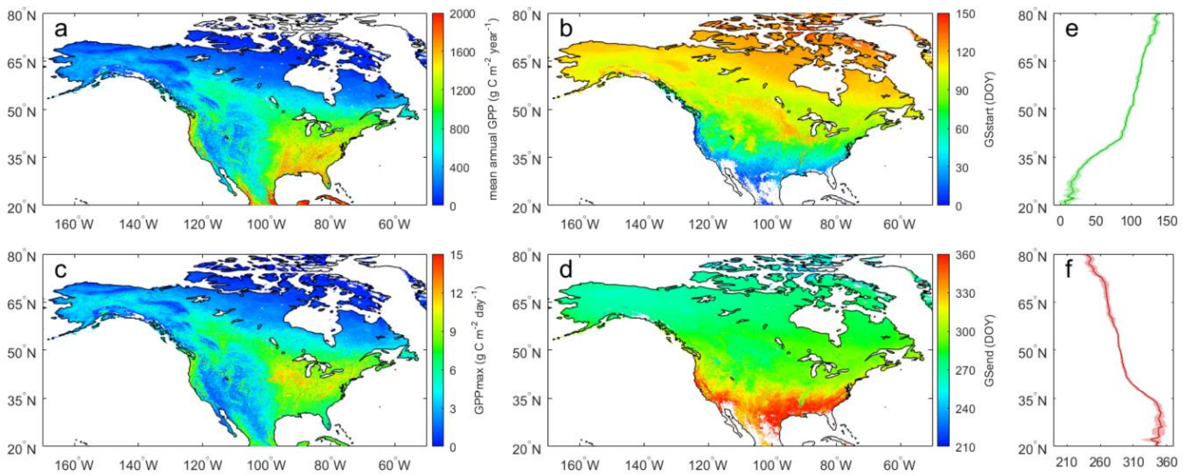

Figure S2. Mean values of annual GPP and the three indicators over the period 2000-2014 across North America. (**a**) Annual GPP. (**b**) GS_{start}. (**c**) GPP_{max}. (**d**) GS_{end}. Latitudinal distributions of (**e**) GS_{start} (green) and (**f**) GS_{end} (red). Maps were generated using MATLAB 2015b (http://www.mathworks.com/products/matlab/).

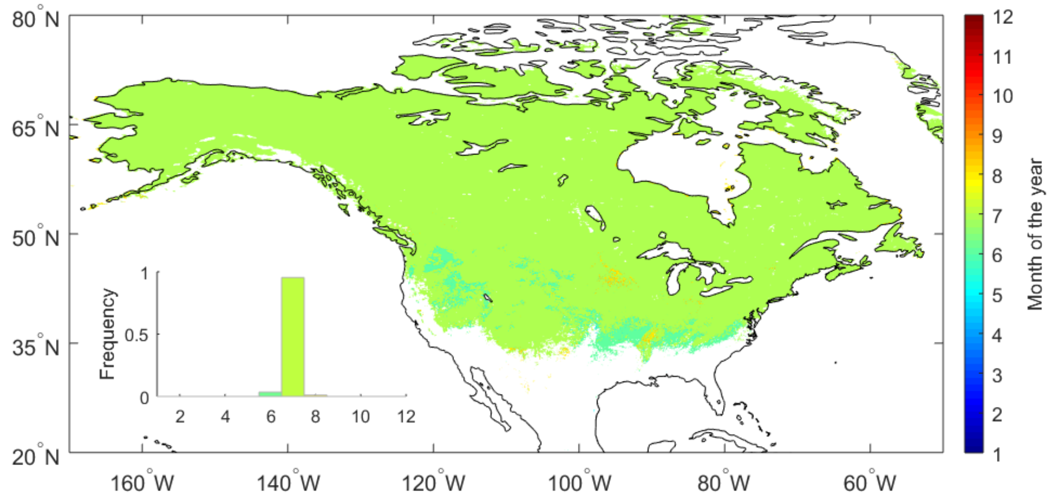

Figure S3. Mean date of GPP_{max} over the period 2000-2014 across North America. Maps were generated using MATLAB 2016a (http://www.mathworks.com/products/matlab/).

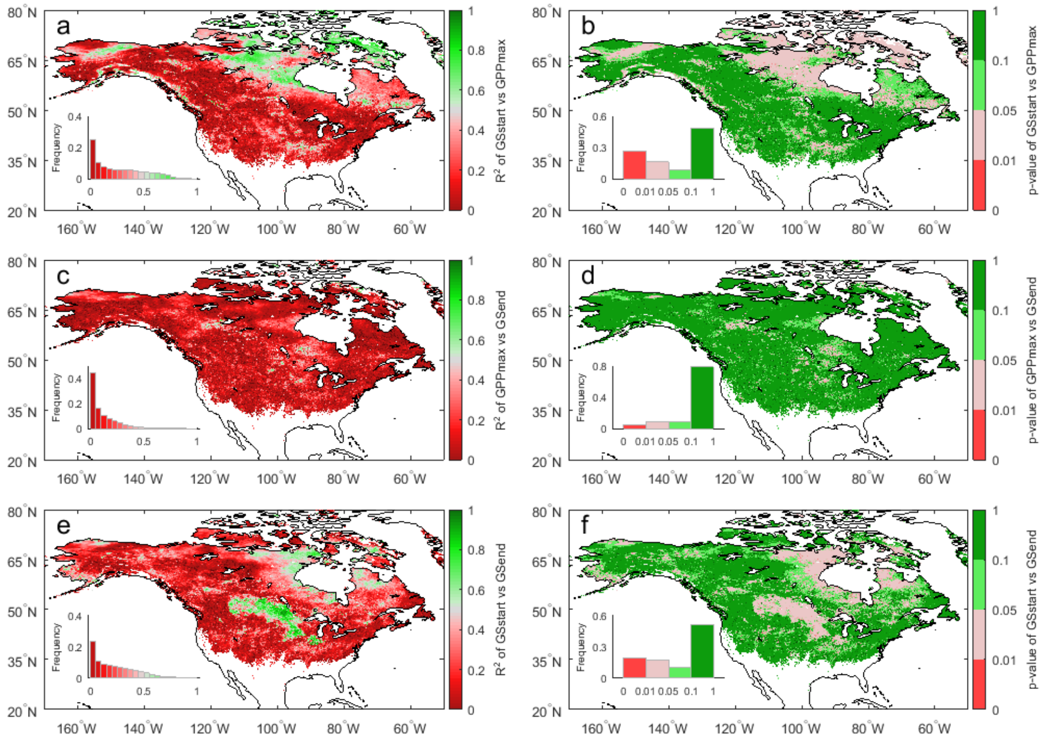

Figure S4. Coefficients of determination (R^2) and significance level (p-value) of the interrelationships of the three indicators. (**a, b**) GS_{start} v.s. GPP_{max}. (**c, d**) GPP_{max} v.s. GS_{end}. (**e, f**) GS_{start} v.s. GS_{end} . Maps were generated using MATLAB 2016a (http://www.mathworks.com/products/matlab/).

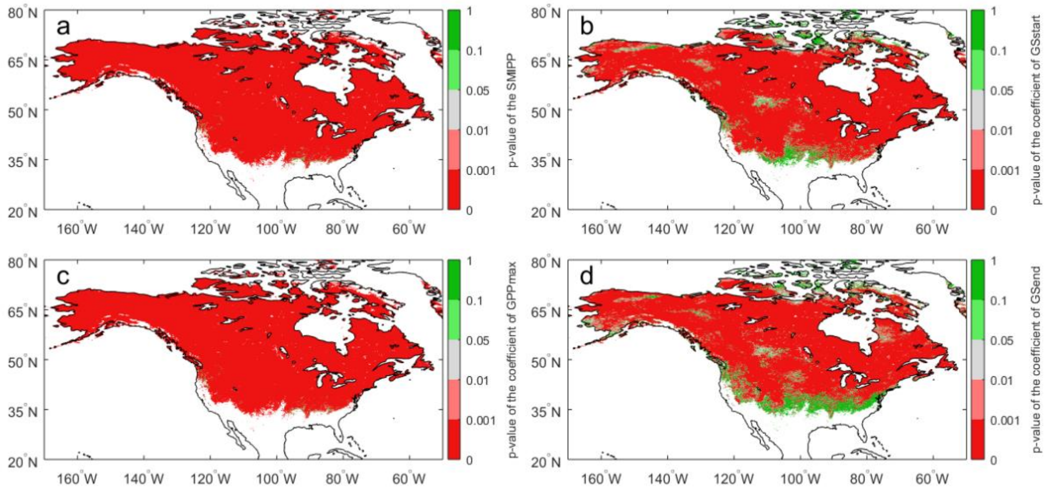

Figure S5. Significance level (p-value) of the SMIPP and the three sensitivity coefficients. (**a**) SMIPP. (**b**) GS_{start}. (**c**) GPP_{max}. (**d**) GS_{end}. Maps were generated using MATLAB 2016a (http://www.mathworks.com/products/matlab/).

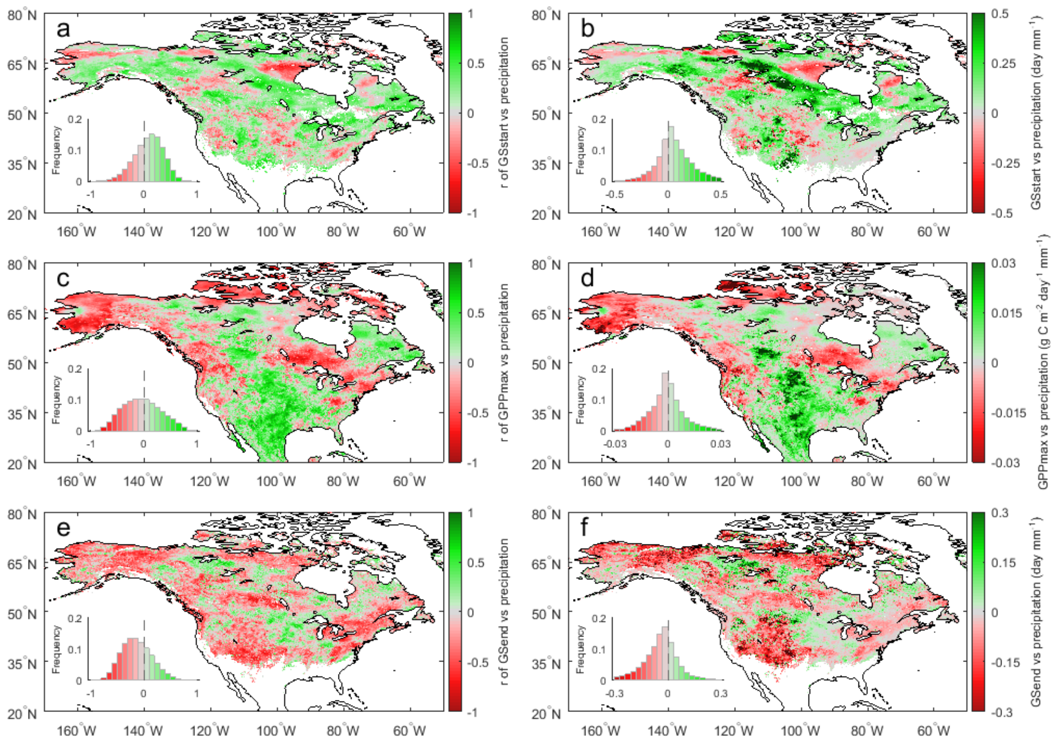

Figure S6. Correlation coefficient (r) and slope between the three indicators and respective seasonal precipitation in North America. (**a, b**) GS_{start} and preseason (30 days) total precipitation. (**c, d**) GPP_{max} and summer precipitation (mean monthly precipitation in June-July-August). (**e, f**) GS_{end} and preseason (30 days) total precipitation. Maps were generated using MATLAB 2016a (http://www.mathworks.com/products/matlab/).

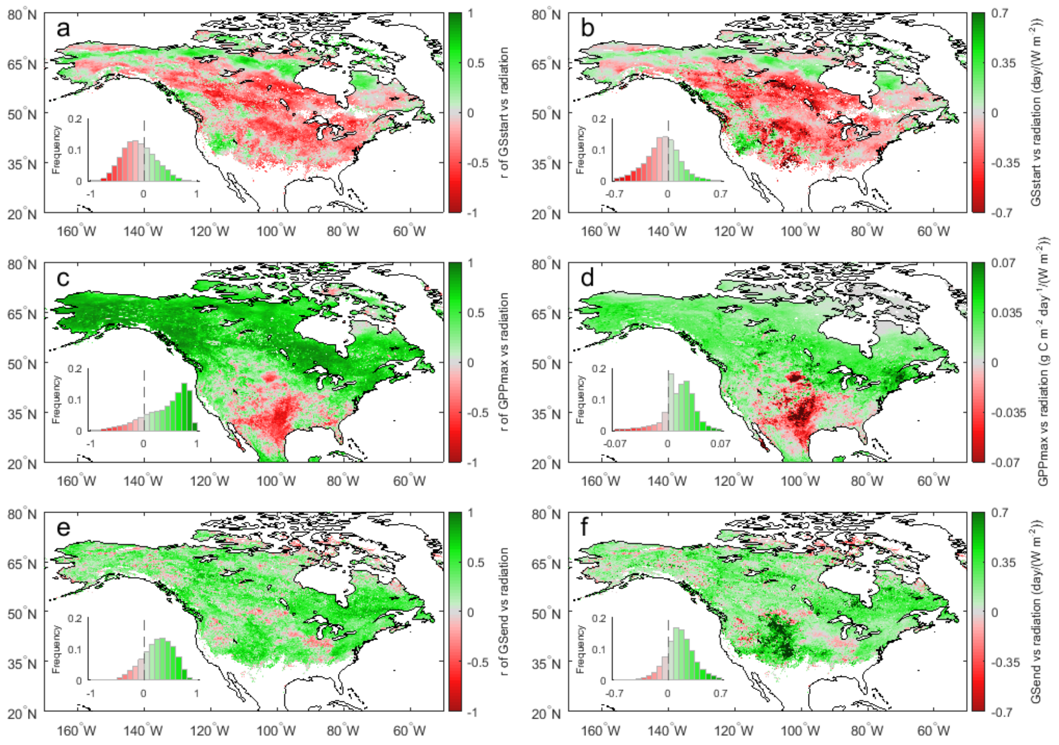

Figure S7. Correlation coefficient (r) and slope between the three indicators and respective seasonal solar radiation in North America. (**a, b**) GS_{start} and preseason (30 days) solar radiation. (**c, d**) GPP_{max} and summer solar radiation (mean solar radiation in June-July-August). (**e, f**) GS_{end} and preseason (30 days) solar radiation. Maps were generated using MATLAB 2016a (http://www.mathworks.com/products/matlab/).

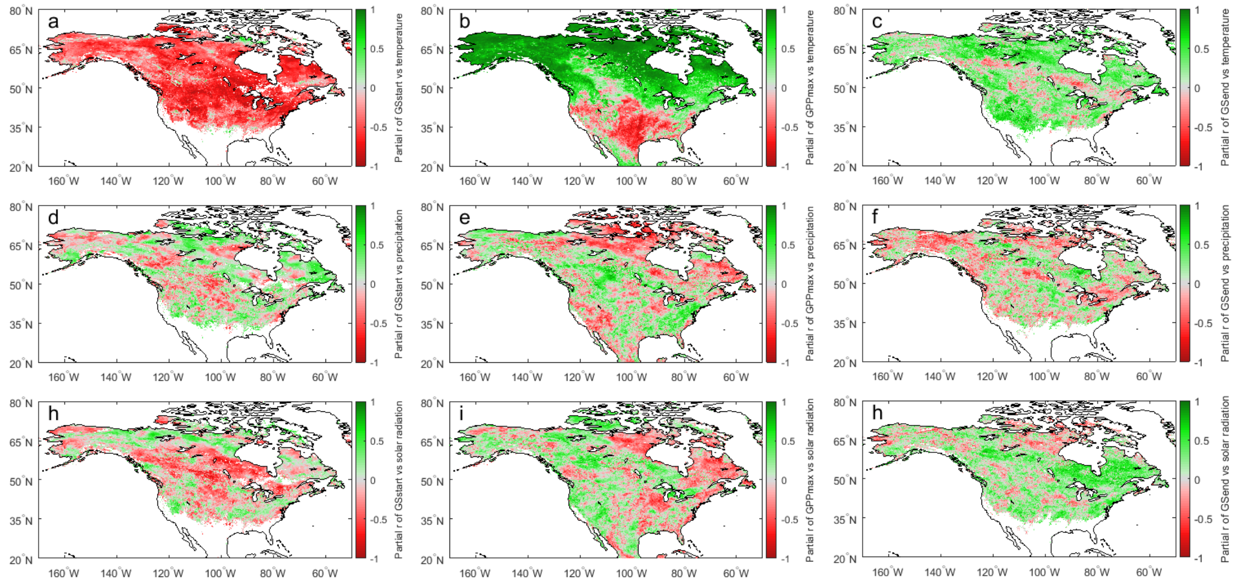

Figure S8. Partial correlation coefficient (r) between the three indicators and respective seasonal climatic factors (temperature, precipitation, solar radiation) in North America. (**a, d, h**) GS_{start} and preseason (30 days) climatic factors. (**b, e, i**) GPP_{max} and summer climatic factors. (**c, f, h**) GS_{end} and preseason (30 days) climatic factors. Maps were generated using MATLAB 2016a (http://www.mathworks.com/products/matlab/).

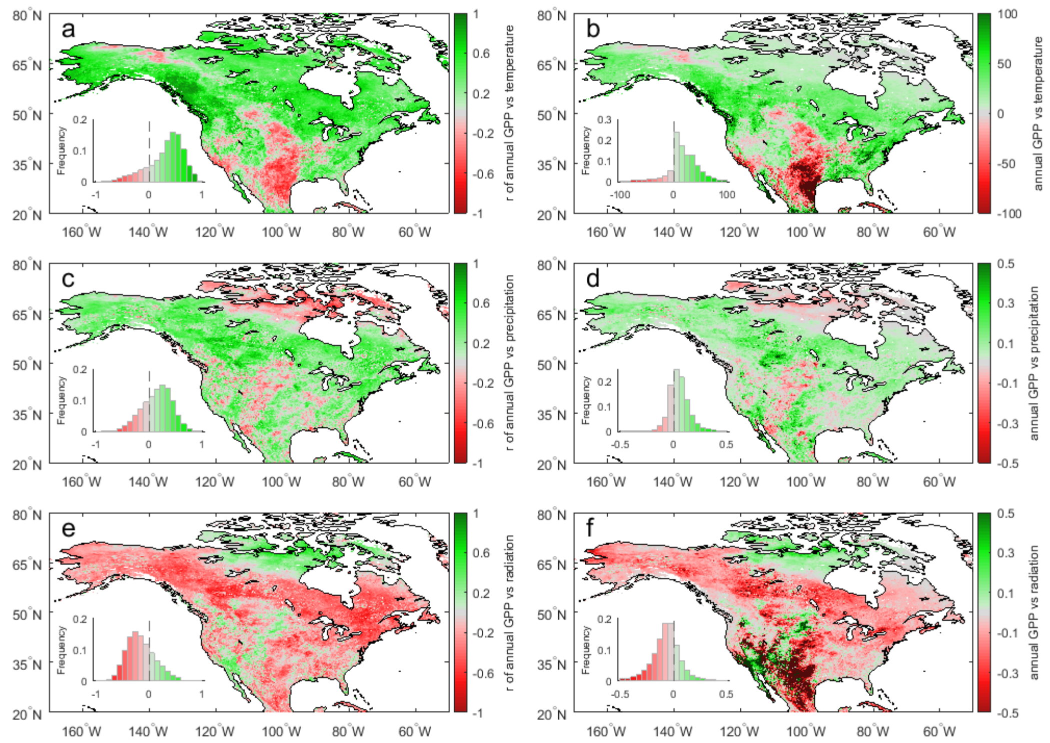

Figure S9. Correlation coefficient (r) and slope between annual GPP and climatic factors. (**a, b**) Annual GPP and mean temperature (g C m^{-2}/°C). (**c, d**) Annual GPP and total precipitation (g C m^{-2} mm^{-1}). (**e, f**) Annual GPP and solar radiation (g C m^{-2}/(W m^{-2})). Maps were generated using MATLAB 2016a (http://www.mathworks.com/products/matlab/).

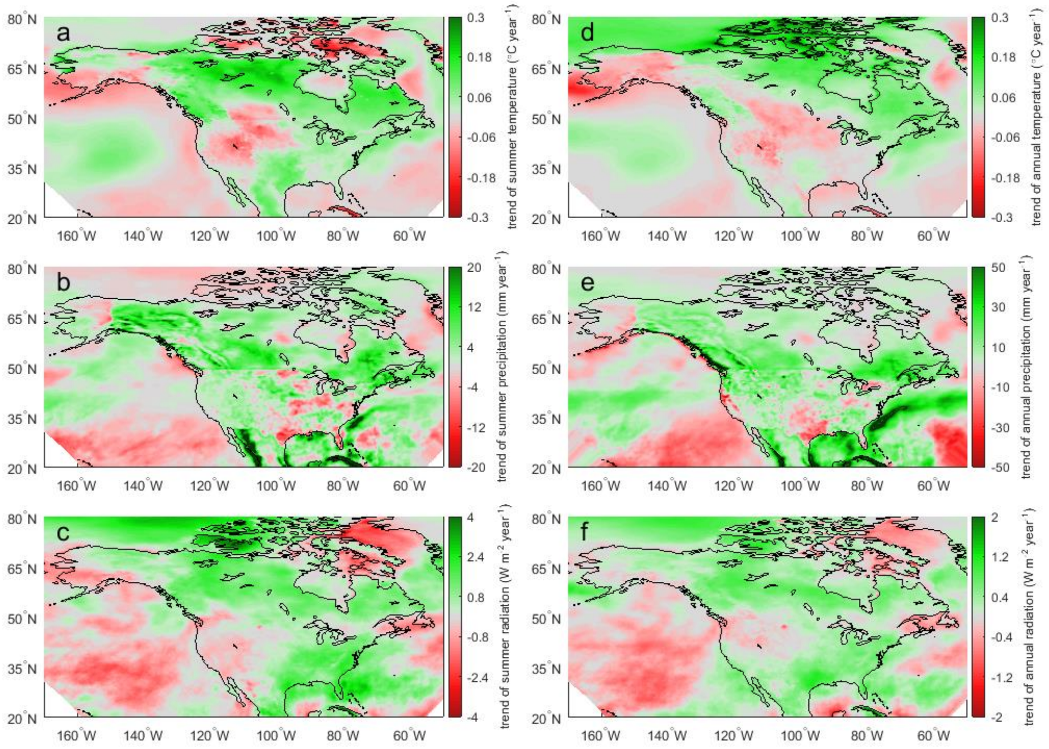

Figure S10. Long-term trends of summer and annual climatic factors over the period 2000-2014 across North America. (**a, d**) Mean temperature. (**b, e**) Total precipitation. (**c, f**) Mean solar radiation. Maps were generated using MATLAB 2016a (http://www.mathworks.com/products/matlab/).

References

1. Hollinger, S. E., Bernacchi, C. J. & Meyers, T. P. Carbon budget of mature no-till ecosystem in North Central Region of the United States. *Agric. For. Meteorol.* **130**, 59-69 (2005).
2. Matamala, R., Jastrow, J. D., Miller, R. M. & Garten, C. T. Temporal changes in C and N stocks of restored prairie: Implications for C sequestration strategies. *Ecol. Appl.*, **18**, 1470-1488 (2008).
3. Suyker, A. E., Verma, S. B., Burba, G. G. & Arkebauer, T. J. Gross primary production and ecosystem respiration of irrigated maize and irrigated soybean during a growing season. *Agric. For. Meteorol.* **131**, 180-190 (2005).
4. Xue, L. & Pan, Z. Ensemble calibration and sensitivity study of a surface CO_2 flux scheme using an optimization algorithm. *J. Geophys. Res. Atmospheres* **113**, D10109 (2008).
5. Liang, S. *et al.* Estimation of incident photosynthetically active radiation from Moderate Resolution Imaging Spectrometer data. *J. Geophys. Res. Atmospheres* **111**, D15208 (2006).
6. Urbanski, S. *et al.* Factors controlling CO_2 exchange on timescales from hourly to decadal at Harvard Forest. *J. Geophys. Res. Biogeosciences* **112**, G02020 (2007).
7. Dragoni, D., Schmid, H. P., Grimmond, C. S. B. & Loescher, H. W. Uncertainty of annual net ecosystem productivity estimated using eddy covariance flux measurements. *J. Geophys. Res. Atmospheres* **112**, D17102 (2007).
8. Noormets, A., McNulty, S. G., DeForest, J. L., Sun, G., Li, Q. & Chen, J. Drought during canopy development has lasting effect on annual carbon balance in a deciduous temperate forest. *New Phytol.* **179**, 818-828 (2008).
9. Clark, K. L., Skowronski, N., Hom, J. & Heilman, W. E. Fireflux Experiments in the New Jersey Pine Barrens, paper presented at Kalispell (MT): Eighth Symposium on Fire and Forest Meteorology, Citeseer (2009).
10. Gough, C.M., Vogel, C. S., Schmid, H. P., Su, H. B. & Curtis, P. S. Multi-year convergence of biometric and meteorological estimates of forest carbon storage. *Agric. For. Meteorol.* **148**, 158-170 (2008).
11. Nave, L. E. *et al.* Disturbance and the resilience of coupled carbon and nitrogen cycling in a north temperate forest. *J. Geophys. Res. Biogeosciences* **116**, G04016 (2011).
12. Yi, C. *et al.* Observed covariance between ecosystem carbon exchange and atmospheric

boundary layer dynamics at a site in northern Wisconsin. *J. Geophys. Res. Atmospheres* **109**, D08302 (2004).

13. Giasson, M. A., Coursolle, C. & Margolis, H. A. Ecosystem-level CO_2 fluxes from a boreal cutover in eastern Canada before and after scarification. *Agric. For. Meteorol.* **140**, 23-40 (2006).
14. Bergeron, O., Margolis, H. A., Black, T. A., Coursolle, C., Dunn, A. L., Barr, A. G. & Wofsy S. C. Comparison of carbon dioxide fluxes over three boreal black spruce forests in Canada. *Global Change Biol.* **13**, 89-107 (2007).
15. Yuan, F. *et al.* Modeling analysis of primary controls on net ecosystem productivity of seven boreal and temperate coniferous forests across a continental transect. *Global Change Boil.* **14**, 1765-1784 (2008).
16. Zeller, K. & Nikolov, N. Quantifying simultaneous fluxes of ozone, carbon dioxide and water vapor above a subalpine forest ecosystem. *Environ. Pollut.* **107**, 1-20 (2000).
17. Sacks, W. J., Schimel, D. S., Monson, R. K. & Braswell, B. H. Model-data synthesis of diurnal and seasonal CO_2 fluxes at Niwot Ridge, Colorado. *Global Change Biol.* **12**, 240-259 (2006).
18. Scurlock, J. M. O., Johnson, K. & Olson, R. J. Estimating net primary productivity from grassland biomass dynamics measurements. *Global Change Biol.* **8**, 736-753 (2002).
19. McCaughey, J., Pejam, M., Arain, M. & Cameron, D. Carbon dioxide and energy fluxes from a boreal mixedwood forest ecosystem in Ontario, Canada. *Agric. For. Meteorol.* **140**, 79-96 (2006).
20. Hollinger, D. Y. *et al.* Spatial and temporal variability in forest-atmosphere CO_2 exchange. *Global Change Biol.* **10**, 1689-1706 (2004).
21. Saito, M., Maksyutov, S., Hirata, R. & Richardson, A. D. An empirical model simulating long-term diurnal CO_2 flux for diverse vegetation types. *Biogeosciences*, **6**, 585-599 (2009).
22. Desai, A. R., Bolstad, P. V., Cook, B. D., Davis, K. J. & Carey, E. V. Comparing net ecosystem exchange of carbon dioxide between an old-growth and mature forest in the upper midwest, USA. *Agric. For. Meteorol.* **128**, 33-55 (2005).
23. Peel, M. C., Finlayson, B. L. & McMahon, T. A. Updated world map of the Koppen-Geiger climate classification. *Hydrol. Earth Syst. Sci.* **11**, 1633-1644 (2007).
